# Incidentally Diagnosed Asymptomatic Crohn’s Disease: A Retrospective Cohort Study of Long-Term Clinical Outcomes

**DOI:** 10.1093/crocol/otac034

**Published:** 2022-09-13

**Authors:** Ana Grinman, Bella Ungar, Adi Lahat, Uri Kopylov, Rami Eliakim, Shomron Ben-Horin

**Affiliations:** Department of Gastroenterology, Sheba Medical Center, Sackler School of Medicine, Tel-Aviv University, Tel Aviv, Israel; Department of Gastroenterology, Sheba Medical Center, Sackler School of Medicine, Tel-Aviv University, Tel Aviv, Israel; Department of Gastroenterology, Sheba Medical Center, Sackler School of Medicine, Tel-Aviv University, Tel Aviv, Israel; Department of Gastroenterology, Sheba Medical Center, Sackler School of Medicine, Tel-Aviv University, Tel Aviv, Israel; Department of Gastroenterology, Sheba Medical Center, Sackler School of Medicine, Tel-Aviv University, Tel Aviv, Israel; Department of Gastroenterology, Sheba Medical Center, Sackler School of Medicine, Tel-Aviv University, Tel Aviv, Israel

**Keywords:** Crohn’s disease, inflammatory bowel disease, complications

## Abstract

**Background:**

Crohn’s disease (CD) is occasionally diagnosed in asymptomatic patients who have undergone colonoscopy or imaging for other reasons. The clinical outcome and optimal management of these patients remain poorly defined.

**Methods:**

This was a retrospective cohort study of asymptomatic patients with incidental diagnosis of CD from the electronic patient registry of the IBD Unit of Sheba Medical Center in Israel. The primary outcome was defined as the occurrence of a clinical flare.

**Results:**

Of the 2700 CD patients in Sheba IBD registry, 60 asymptomatic patients with incidental diagnosis of CD were identified (31/60 males, median age 50.5, 25%–75% interquartile range [IQR] 43.5–57.25 years, median follow-up 4.5 years, 25–75% IQR 2.5–6.75, range 1–15 years). Most of the patients did not receive any treatment after diagnosis (53/60—88.33%). Of these, 5 patients (9.43%) experienced a flare during follow-up (median 4.5 years, IQR 2.5–6.75, range 1–15 years). Patients with subsequent flare had numerically higher CRP at diagnosis than patients who did not flare (2.2, IQR 2.0–3.0 vs 1.04, IQR 1.0–2.2, *P* = .09). When comparing the group of patients who received treatment immediately after diagnosis (*n* = 7) with the group who did not receive treatment (*n* = 53), there was no difference with respect to the survival time without a flare (*P* = .3). For other secondary outcomes, 3/40 progressed from B1 phenotype to B2, and 3/53 (6%) patients underwent surgery during the follow-up.

**Conclusions:**

The majority of patients with an incidental diagnosis of asymptomatic CD can probably be followed-up without immediate treatment. Although most remain asymptomatic and without complications during follow-up, close monitoring for disease progression is prudent.

## Introduction

Diagnosis of Crohn’s disease (CD) is usually made at a symptomatic stage when patients seek medical help due to symptoms such as diarrhea, abdominal pain, rectal bleeding, and weight loss. In such cases, once the diagnosis is established, treatment is initiated in order to control inflammatory activity, ameliorate symptoms, and prevent disease progression.

Occasionally however, asymptomatic patients may be incidentally diagnosed with CD after undergoing colonoscopy or imaging studies for reasons unrelated to clinical symptoms of CD.^1^

The question that arises in such cases pertains to the management and follow-up of these patients: Specifically, is treatment mandatory and how would they evolve without treatment? For asymptomatic patients diagnosed incidentally this question is especially pertinent given that most therapeutic agents are not exempt from risks and adverse effects.

Only a few studies reported on the natural history and outcome of patients with CD who were asymptomatic upon an incidental diagnosis, mostly found during screening colonoscopy.^1,2^ However, only a minority of patients in these reports were patients with CD and the outcomes of untreated patients were largely missing,^1,2^ thereby rendering as yet undefined the long-term outcome of such patients without any treatment.

The present study therefore aimed to evaluate the natural history, disease progression, and the need for treatment in patients with asymptomatic CD, which was diagnosed incidentally.

## Methods

### Design and Patient Population

This was a retrospective cohort study. The IBD database of the Gastroenterology Department of the Sheba Medical Center, a tertiary academic center in Israel, was scanned for Crohn’s patients who were initially diagnosed incidentally in the absence of clinical symptoms, while undergoing colorectal screening colonoscopy, work-up for symptoms unrelated to inflammatory bowel disease (IBD) (see definitions below) or for asymptomatic iron deficiency anemia.

Demographic and clinical data were retrieved from the electronic medical records of identified patients, including age, sex, body mass index, disease duration, hemoglobin, CRP, albumin and calprotectin at diagnosis, smoking status, ethnicity, presence of comorbidities, and Montreal classification. In all patients, the diagnosis of CD was made based on endoscopic and radiologic findings and confirmed with compatible histopathology.^3^

Exclusion criteria were age <18, unconfirmed CD or loss to follow-up immediately after the diagnosis.

### Definitions and Outcomes

We defined asymptomatic CD at diagnosis as patients who were either completely asymptomatic and underwent screening colonoscopy, or patients whose symptoms were considered irrelevant and unrelated to CD at diagnosis. Irrelevant symptoms were pragmatically defined as symptoms not ascribed to the incidentally found CD and for which other etiologies were considered the more plausible cause (eg, rectal bleeding in a patient with hemorrhoids and isolated small bowel CD, constipation in a patient with small bowel CD and so forth). Asymptomatic iron deficiency anemia, that is, without weakness, dizziness or fatigue on exertion, or a positive occult blood test were also acceptable for the definition of a clinically asymptomatic CD, and treatment with iron supplementation alone was not considered as CD treatment for this study.

Clinical flare was defined as onset of symptoms related to active CD as per the judgment of the treating physician, which could not be explained by other etiologies and coupled with objective flare signs, which were defined as elevation of serum CRP above the upper limit of normal and/or fecal calprotectin above 100 mcg/g or endoscopic or radiologic evidence of disease activity.

The primary outcome was the time to clinical flare in patients with asymptomatic CD at diagnosis who did not receive any treatment. Secondary outcomes included time to clinical flare, complications (phenotype progression) or surgery, and factors related to the risk of clinical flare in these patients as well as the comparison of the primary outcome (time to flare) among patients who have not received treatment with those who have.

The study was approved by the Institutional Ethics Review Board and was exempted from patients’ consent due to its retrospective chart review nature.

### Statistics

Descriptive statistics were employed using median and interquartile ranges (IQRs). Survival analysis was computed for the outcome of interest using Kaplan–Meier curves. Comparisons between subgroups were performed using Fisher’s exact test or Mann–Whitney *U*-test for categorical and continuous variables, respectively. All reported *P* values were 2-sided, and a *P* value less than .05 was considered statistically significant. All statistical calculations were performed with the use of MedCalc software (version 12.2.1.0).

## Results

### Study Population

Of the 2700 Crohn’s patients in Sheba IBD registry at study inception, 62 patients who were asymptomatic at diagnosis were identified. Two were excluded due to lack of any follow-up after the initial diagnosis. Thus, the study included 60 asymptomatic patients with incidental diagnosis of CD on colonoscopy, performed due to other indications (including an incidental suspicion of CD on imaging studies). The baseline characteristics of the patients included are shown in Table 1. As shown, the median age was 50.5, and most, but not all of the patients had B1 inflammatory phenotype. Most of the patients included were not given any treatment for their CD upon diagnosis (53/60—88.33%).

**Table 1.** Clinical and demographics characteristics of the study cohort.

Clinical–demographic parameters	Number (percentage)
Males, *n* (%)	31 (51.6)
Age at diagnosis, median (IQR), year	50.5 (43.5–57.25)
BMI, median (IQR)	23.85 (22.1–26.82)
Smoking, *n* (%)	
Never smokers	44 (73.3)
Ex-smokers	1 (1.6)
Current smokers	5 (8.33)
Unknown status	10 (16.6)
Follow-up, median (IQR), year	4.5 (2.5–6.75)
Disease location (Montreal classification)	
L1	37 (61.66)
L2	9 (15.0)
L3	13 (21.66)
L4	1 (1.66)
Perianal disease	1 (1.66)
Disease behavior (Montreal classification)	
B1	46 (76.6)
B2	12 (20.0)
B3	2 (3.33)
Indication for endoscopy	
Screening	19 (31.66)
Imaging finding	4 (6.66)
Anemia	8 (13.33)
Rectal bleeding (in small bowel CD)	10 (16.66)
Fecal occult blood positive	6 (10.0)
Other^a^	13 (21.66)
NSAIDS use at diagnosis (%)	8 (13.33)

Abbreviations: BMI, body mass index; CD, Crohn’s disease; EIM, extra intestinal manifestations; EN, erythema nodosum; IQR, interquartile range; NSAIDs, nonsteroidal anti-inflammatory drugs.

Iron deficiency without anemia, anal pruritus.

For the primary outcome, among the 53 patients who did not receive any medical treatment at diagnosis, 5 patients (9.43%) had a clinical flare over the follow-up period (median follow-up 4.5 years, 25–75% IQR 2.5–6.75, range 1–15 years). Clinical details of the 5 patients who flared during follow-up are shown in Table 2.

**Table 2.** Clinical characteristics of patients not treated after initial diagnosis with a clinical flare during follow-up.

Gender	Age at diagnosis	Endoscopy indication	Montreal classification at diagnosis	Follow-up until flare (years)	Signs and symptoms of flare	Treatment initiated for flare
M	54	Screening	B3 L3	2	Elevated calprotectin and CRP, abdominal pain, diarrhea	Infliximab
M	50	Screening	B1 L3	12	Rectal bleeding and cecum ulceration at colonoscopy	Budesonide
M	21	Rectal bleeding	B2 L1	1	Elevated CRP, abdominal pain, subocclusion symptoms	Prednisone, azathioprine, adalimumab
F	53	Screening	B1 L3	6	Elevated CRP, abdominal pain, diarrhea, arthritis	Prednisone, sulfasalazine
F	32	Anemia	B2 L3	1	Elevated CRP, abdominal pain	Adalimumab

Seven patients received treatment immediately after diagnosis despite being asymptomatic comprising the following agents or a combination: mesalazine (*n* = 4), budesonide (*n* = 3), azathioprine (*n* = 1), and prednisone (*n* = 1).

**Figure 1.**
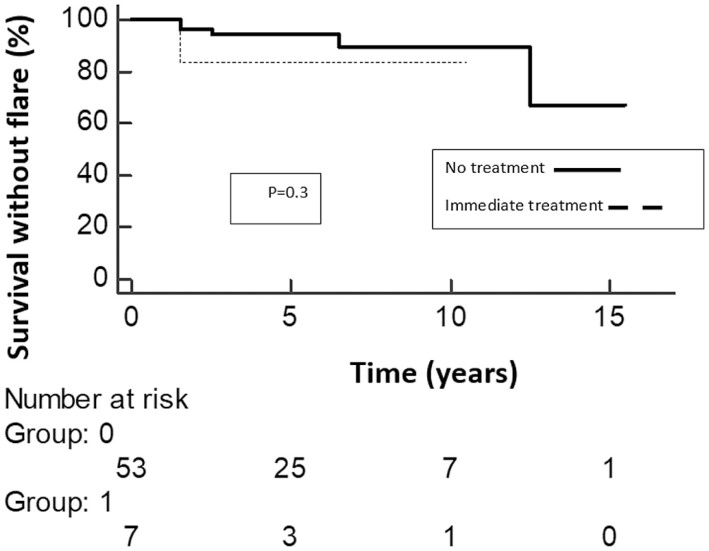
Survival time without a clinical flare in patients not receiving any treatment, and compared to patients immediately initiating postdiagnosis treatment.

When comparing the group of patients who received treatment immediately after diagnosis with the group of patients who did not receive treatment at diagnosis, there was no statistically significant difference in the survival time without flare (*P* = .3, Figure 1).

**Figure 2.**
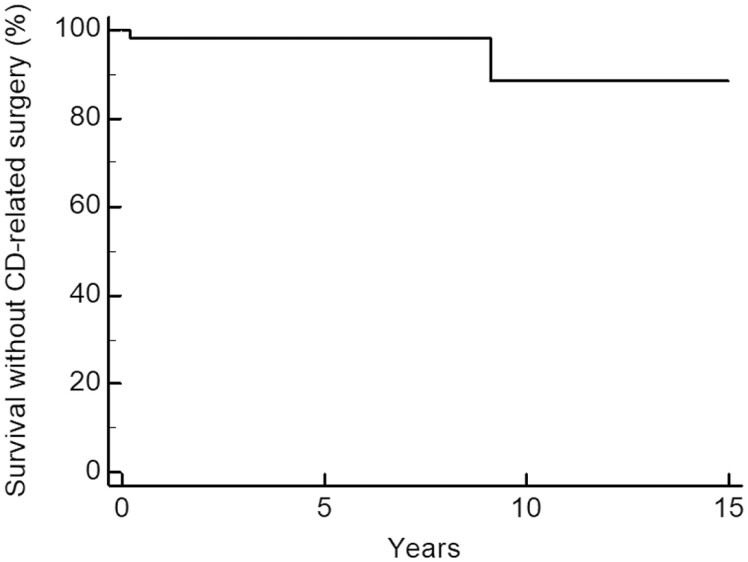
Survival time without CD-related surgery in patients not receiving any treatment. Abbreviation: CD, Crohn’s disease.

For other secondary outcomes, surgery occurred in 3/53 patients (6.0%) after a median of 41 months follow-up after diagnosis (25–75 IQR 2–109, Figure 2). Two of these 3 patients were operated for a colorectal cancer: One 75 years-old man with isolated ileal CD was operated for sigmoid colon carcinoma and may therefore not be truly considered a CD-related surgery. An additional 84 years-old female with ascending colon carcinoma and associated patchy colitis at presentation, which did not receive any treatment during subsequent 5 years of follow-up until she expired from unrelated disease. The third patient was operated after developing a clinical flare with a colonic stenosis. Overall therefore, the secondary outcome of surgery related to CD has occurred in 2/53 (3.7%) of the cohort.

At diagnosis, 46 patients were classified as phenotype B1 by the Montreal classification, 12 as B2 and 2 as B3. Of the 40 patients with B1 initial phenotype and data available on phenotype at end of follow-up, 3 patients (7.5%) evolved to a B2 stricturing phenotype (1 with clinical flare, colon stenosis, and surgery and 2 without a clinical flare or surgery).

We examined several factors for their potential utility to predict the risk of flare during follow-up.

**Figure 3.**
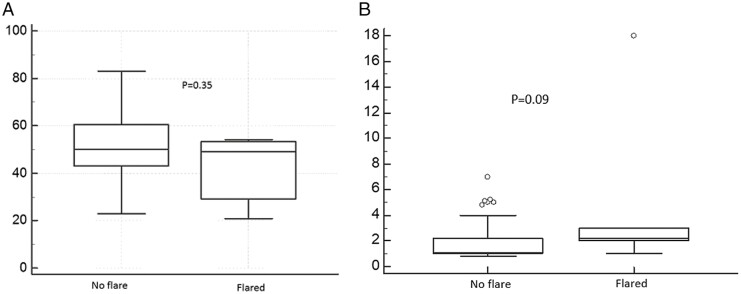
(A) Median age at diagnosis of patients with or without a clinical flare during follow-up. (B) Median CRP levels at diagnosis in patients with or without a clinical flare during follow-up.

For most parameters examined, including age, ileal versus other locations, or having a complicated phenotype (B2/B3 vs B1) were not found to predict a clinical flare later on (Figure 3A). A high level of CRP at diagnosis was found to have a trend, which did not reach statistical significance, to predict a clinical flare later on (median CRP 2.2, IQR 2.0–3.0 versus median CRP 1.04, IQR:1.0–2.2, *P* = .09, Figure 3B).

## Discussion

The present study examined the outcome of patients with incidentally diagnosed CD and no immediate treatment after diagnosis. The results indicate the majority (over 90%) of these patients did not present a clinical flare or progressed during a relatively long follow-up (some more than 10 years). Additionally, there was no difference in outcomes between patients who did not receive treatment immediately after diagnosis and patients who did (*P* = .3), although this comparison was limited by a small number of patients in the treatment group.

In a study published only in an abstract form, Cosnes et al analyzed a group of 43 patients with asymptomatic CD at diagnosis (silent CD) and compared it with the recurrence of postoperative patients and clinically active patients.^4^ During a median follow-up of 78 months (range 3–216) 72% of the silent CD group developed symptoms after a median time of 46 months (2–109), and 10 developed CD complication and required surgical resection. The evolution was similar in the postoperative group but was significantly more severe in the clinically active group. The authors concluded that the natural history of silent CD reproduces the model of postoperative recurrence from endoscopic lesions to symptoms and complications.^4^ Our study encompassed a somewhat larger size cohort and does not recapitulate the French observations entirely since the ensuing course of our patients was relatively benign. Only 5/53 untreated patients experienced a clinical flare during a relatively long-term follow-up. Moreover, only 3 patients required surgery, of whom 2 required a surgery due to colorectal cancer, which one was probably unrelated to his isolated small bowel CD. In another study reported only in abstract form, 55 IBD patients were incidentally diagnosed during a screening colonoscopy program in the United Kingdom, of whom 16 had CD. Only 20 patients were reported to be asymptomatic and 7/20 (35%) became symptomatic during follow-up, but there was no detailed description of the UC/CD diagnosis and of the outcomes for these 7 patients except that none required surgery.^1^

A recent systematic review by Agrawal et al examined the entity designated by the authors as “incidentally diagnosed terminal ileitis” (IDTI) during colonoscopy performed for unrelated reasons, also termed isolated active ileitis.^5^ They have found that progression of these patients to overt CD or histologic proof of CD was rare. A similar result was seen by Tse et al^6^ in a retrospective study of 108 patients with isolated acute terminal ileitis. Whether these patients comprise a different disease entity that should be designated IDTI or are they simply silent CD is hard to definitively establish. However, the clinical outcome of our patients—regardless of the terminology employed—is more in agreement with the data of the Agrawal study than the Cosnes one. Contrary to the Agrawal study however, histologic features of CD were present in all our patients, of whom 20/48 had disease involving the colon, and not merely an isolated small bowel disease, further supporting the diagnosis of genuine silent CD in our cohort.

In another study, 40 patients with ileal lesions were identified from a cohort of patients undergoing screening colonoscopy.^7^ Of the 13/40 patients whose diagnosis was confirmed as CD, 5/13 received treatment, thereby limiting the interpretation of natural outcome of untreated incidentally diagnosed CD.

IBD is preceded by an asymptomatic preclinical period. During this phase, in the absence of symptoms and bowel lesions, there are already pathogenic mechanisms comprising dysregulation of the intestinal immune system, microbial perturbation, epithelial barrier permeability alterations, and activation of intestinal inflammatory process. What initially leads to this activation and deregulation of the immune system and the etiology of the IBD is still a matter of controversy. The Genetic, Environmental, Microbial (GEM) Project prospectively evaluated the development of CD in first-degree relatives of known CD patients and found that healthy persons who eventually developed CD had abnormal LacMan ratios (Lactulose Mannitol Ratio) when enrolled in the study, in some cases more than 3 years prior to development of CD.^8^ However, the GEM study showed that host serum antibody response to 6 microbial antigens in healthy individuals were an early predictor of subsequently developing clinical CD, independent of biomarkers of gut barrier dysfunction, subclinical inflammation, or genetic profile.^8^ This is at least partly in agreement with a study by Torres et al which identified a panel of serum antibodies and proteins that were predictive of healthy individuals who will later develop CD within 5 years, in contrast with UC for which no predicting biomarkers associated with future diagnosis of ulcerative colitis were identified.^9^

Whether our cohort of incidentally diagnosed CD patients is merely early CD patients who will later evolve to “regular” symptomatic CD or are they a unique subgroup (or subphenotype) of patients with long-term indolent course is hard to establish. In patients diagnosed with symptomatic disease, recent studies did show a portion of patients to run an indolent course with little progression.^10^ Notably, almost 25% of our patients had relatively advanced disease (B2/B3 phenotype) upon diagnosis, yet even these remained largely symptom-less and without apparent disease progression throughout several years of follow-up despite receiving no specific treatment. Why these patients are asymptomatic is unclear but it is clear that at least some may have altered perception of pain,^11^ as evident from the silent nature of disease even in the B2 and B3 phenotype patients in our cohort.

Collectively, the present results expand our understanding of the diverse behavior phenotypes of CD, with possible distinction between high risk early aggressive disease which merits early intensive therapy, indolent disease,^10^ and asymptomatic incidentally diagnosed CD that may run an even more indolent course in the majority of patients.

In studies of CD patients in remission, a worse subsequent prognosis was found in those with elevated CRP at the index clinic visit.^12^ In our study, we also saw a trend for higher CRP levels to be associated with subsequent clinical flare during follow-up. In contrast, none of the other factors were found in our study to stratify patients into patients who remained symptom-less and those who flared. This may again suggest that incidentally diagnosed patients are inherently different from symptomatic patients who entered remission, but this finding may merely be secondary to the low event number and under-power to detect future flare predictors. Notwithstanding, to our knowledge, this is the largest study to date reporting the evolution and outcomes of asymptomatic untreated patients after incidental diagnosis of CD. The follow-up of more than 8 years in some patients is another strength of the current study. However, our study has several limitations including the absence of protocolized follow-up culminating in different monitoring strategies employed for these patients by different physicians. Another limitation is that due to the small number of patients that progressed during the follow-up we could not notice any specific imaging features that increased likelihood of progressive disease and future studies are needed.

In conclusion, many asymptomatic patients with an incidental diagnosis of CD can probably be followed-up without immediate treatment. Although the majority remain asymptomatic and without complications during follow-up, close monitoring for disease progression is prudent.

## Data Availability

The data that support the findings of this study are available on request.
